# An Unusual Case of Type 2 Fronto-Ethmoidal Mucopyocele

**DOI:** 10.7759/cureus.29707

**Published:** 2022-09-28

**Authors:** Aishwarya Vijayappan, Prasad Deshmukh, Sagar S Gaurkar, Arjun Panicker, Senu Sunnychan

**Affiliations:** 1 Otolaryngology, Jawaharlal Nehru Medical College, Datta Meghe Institute of Medical Sciences, Wardha, IND; 2 Otolaryngology - Head and Neck Surgery, Jawaharlal Nehru Medical College, Datta Meghe Institute of Medical Sciences, Wardha, IND; 3 Otolaryngology - Head and Neck Surgery and Surgical Oncology, Jawaharlal Nehru Medical College, Datta Meghe Institute of Medical Sciences, Wardha, IND

**Keywords:** endoscopic marsupialization, diagnostic nasal endoscopy, functional endoscopic sinus surgery, pyogenic cyst, cystic lesions, frontal sinus, paranasal mucoceles

## Abstract

Mucoceles of the paranasal sinuses are epithelium-lined cystic masses that develop when the sinus ostia get obstructed. They most frequently occur in the frontal and ethmoid sinuses. The paranasal sinus mucoceles' proximity to the orbit and skull base renders the patient at risk for substantial morbidity. Mucoceles have reactive bone growth, bleeding, fibrosis, and granulation tissue, which are histological traits of respiratory mucosa. The conventional therapy is surgical excision, with endoscopic procedures becoming more popular. A 60-year-old female patient reported to the ENT outpatient clinic complaining of swelling over the medial aspect of her left eye that had begun slowly and progressed over a year. Although there were no neurological, ocular, nasal, or facial symptoms clinically, radiological and cytological examinations aided us in arriving at the definitive diagnosis. The patient in this scenario had an infected mucocele and left medial canthal swelling with no visual impairment, which made it challenging to reach an accurate diagnosis. However, radiological evaluation and cytological examination focused on establishing a definitive diagnosis.

## Introduction

Paranasal mucoceles are progressively developing cystic lesions with pseudostratified columnar epithelium against a background of chronic inflammation filled with inspissated mucus exerting pressure on the normal boundaries of the sinus due to the obstruction of sinus ostium [1]. The mucoceles are frequently packed with thick mucoid secretions that range from clear to yellowish [2]. Congenital defects, allergies, illnesses, trauma, surgical interventions in the nose, and neoplasms can all induce blockage [3]. The mucocele's pressure can lead the sinus to expand, the bone wall to weaken, and the nearby essential structures, such as the orbit and cranial cavity, to extend through the weakest point [2]. Most cases are diagnosed in patients aged 40-60 years, with an almost similar incidence between males and females [4]. The diagnosis is established by medical history, physical exam, and radiological findings. The preferred radiological study is a non-contrast CT of the paranasal sinuses to detect bone erosion and determine mucocele extensions; however, MRI is necessary to differentiate a mucocele from other lesions. If mucoceles are allowed to expand, they can lead to significant morbidity. Such advanced mucoceles present a challenge in their surgical management.

## Case presentation

**Figure 1:**
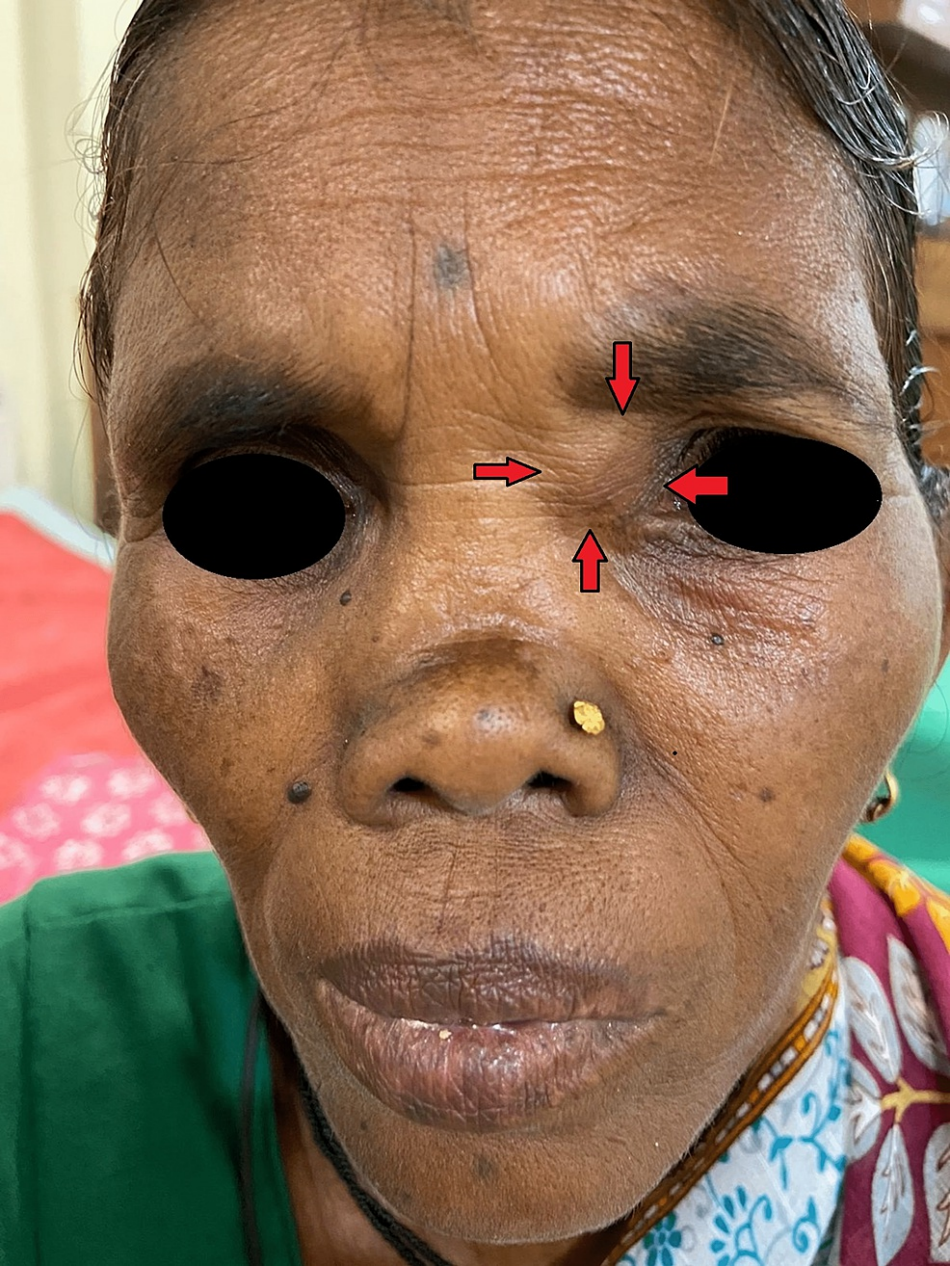
Image showing swelling near medial canthus left eye and nasal deformity

A 60-year-old female patient presented to the ENT outpatient clinic with swelling over the medial portion of her left eye for over a year, which had been insidious in onset and progressive (Figure 1). The swelling was not associated with discharge, pain, itching, or redness in the left eye, and she had no complaints of facial pain or swelling. There was no prior history of diplopia or decreased vision in the left eye. Also, there was no record of nasal obstruction, discharge, or anosmia. Additionally, there were no reports of any history of trauma to the nose, fever, headaches, or giddiness. On physical examination of the nose, a depressed nasal bridge was observed externally. Swelling of 1 x 1 cm was present over the left medial canthus with well-defined margins, soft in consistency, non-tender, and with no local rise in temperature. No paranasal sinus tenderness was found.

**Figure 2:**
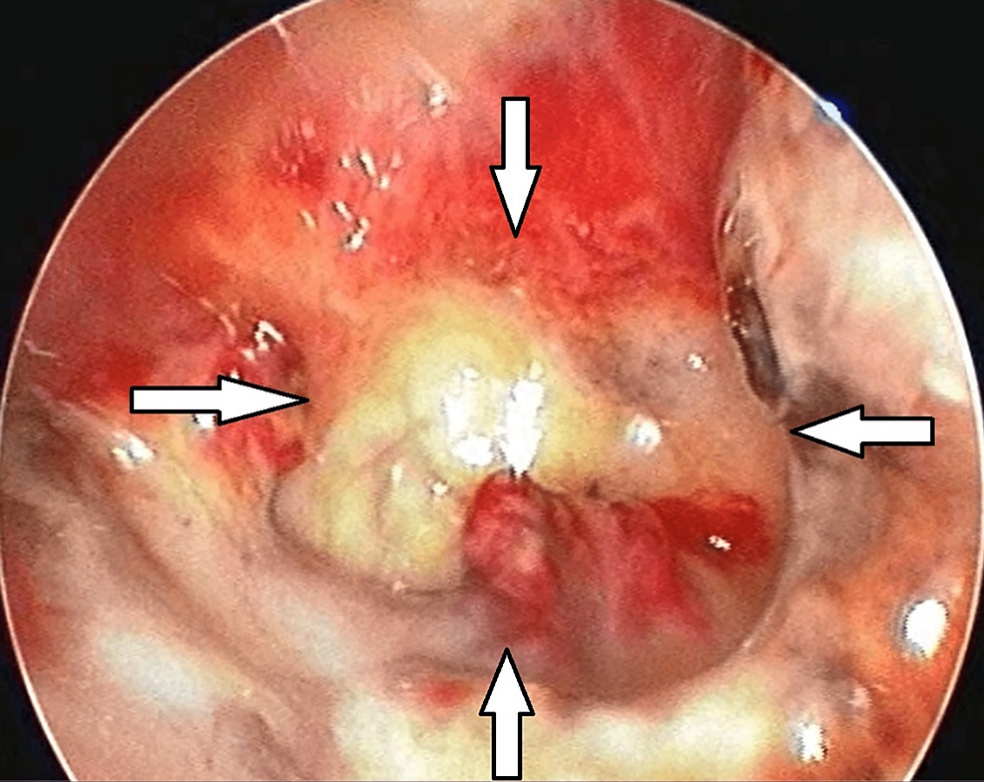
Endoscopy showing a reddish mass in the left nasal cavity (arrows)

An anterior rhinoscopy revealed a roomy nasal cavity with bilateral inferior turbinates atrophy, and a smooth reddish nasal mass was seen arising from the lateral nasal wall. On probe test, the swelling was non-friable, non-tender, and sensitive to touch; attachment of the nasal mass could not be ascertained. Posterior rhinoscopy and nasal patency tests were normal. Ear, throat, neck, and cranial nerves examination was within normal limits. Diagnostic nasal endoscopy disclosed a reddish mass in the left nasal cavity arising from the lateral nasal wall, not bleeding on manipulation and non-friable (Figure 2). It was suggestive of left-side-superior, middle, and inferior turbinate atrophy with visible sphenoid ostium and no evidence of any debris, discharge, or remnant infection. A bulging was seen over the anterior end of the inferior turbinate.

On ophthalmic evaluation, there was no visual impairment and proptosis clinically.

**Figure 3:**
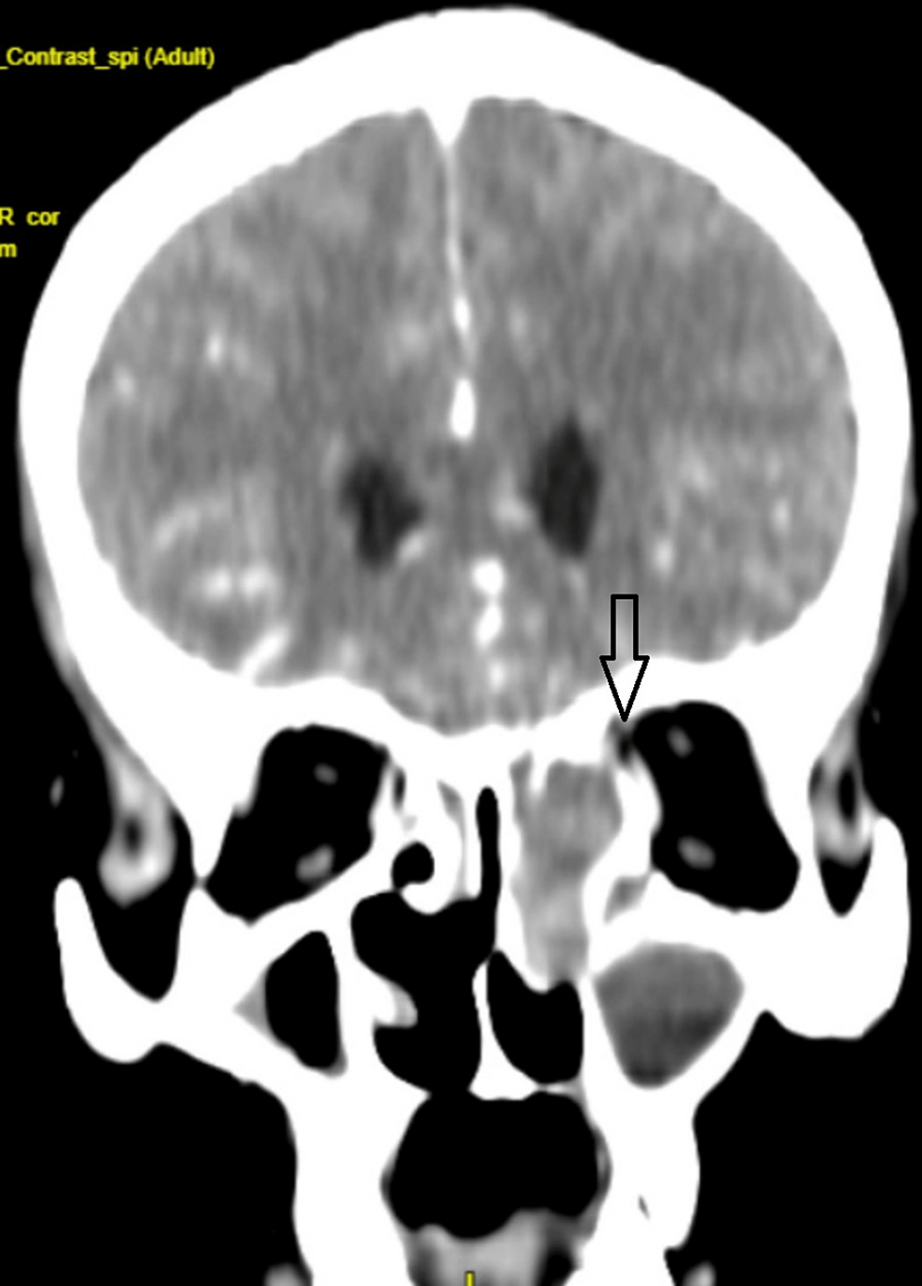
CECT paranasal sinus showing a cortical break in the medial wall of the left orbit (arrow) CECT: contrast-enhanced computed tomography

On contrast-enhanced CT (CECT), there was a heterogeneously enhancing soft tissue density lesion in the left frontal sinus that extended into the ethmoidal and maxillary sinus, causing a widening of the ostium and remodeling with areas of cortical break of the inner plate of the frontal sinus, ethmoidal septa, and medial wall of left orbit with extension into the extraconal compartment of left orbit abutting on medial rectus (Figure 3).

**Figure 4:**
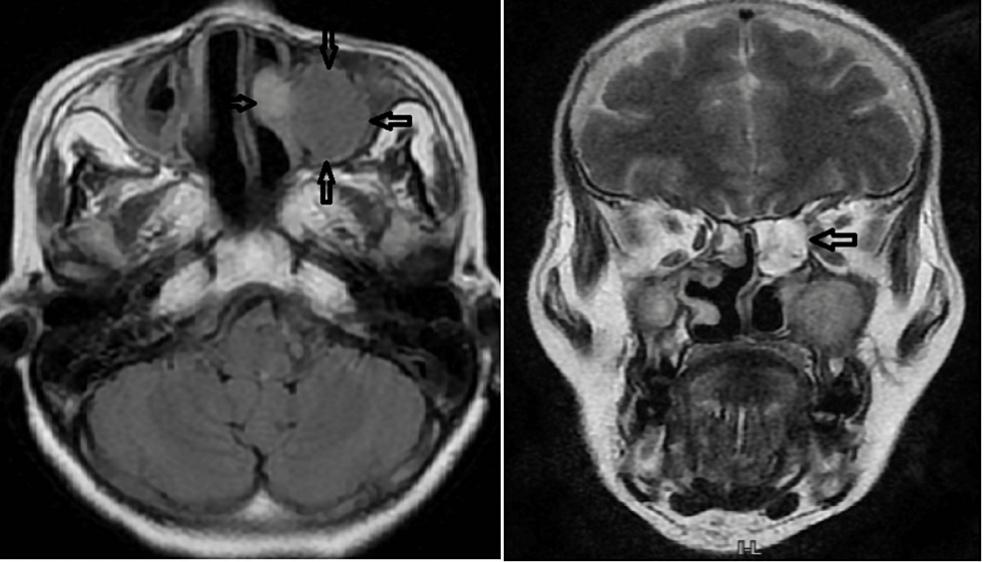
Image showing involvement of left maxillary, frontal, and ethmoid sinuses with expansions The swelling showed extension into the ipsilateral nasal cavity with obstruction of the osteomeatal complex and frontal sinus outflow tract, widening of the infundibulum and ostium with involvement of middle and inferior turbinates

On MRI (Figure 4), a heterogeneous lobulated altered signal intensity lesion appeared isointense on T1, iso to hyperintense on T2, mildly hyperintense on fluid-attenuated inversion recovery (FLAIR) with no enhancement on contrast, showing restriction on diffusion-weighted imaging (DWI). In the left maxillary sinus, there was no blooming on gradient echo (GRE), involving the left maxillary, frontal, and ethmoid sinuses with an expansion of the respective sinuses. The swelling showed extension into the ipsilateral nasal cavity with obstruction of the osteomeatal complex and frontal sinus outflow tract, widening of the infundibulum, and ostium noted with involvement of middle and inferior turbinates. The distended left ethmoid sinus was causing extrinsic compression over the extraconal compartment of the left eye with displacement and mild proptosis of the left eyeball.

Management

**Figure 5:**
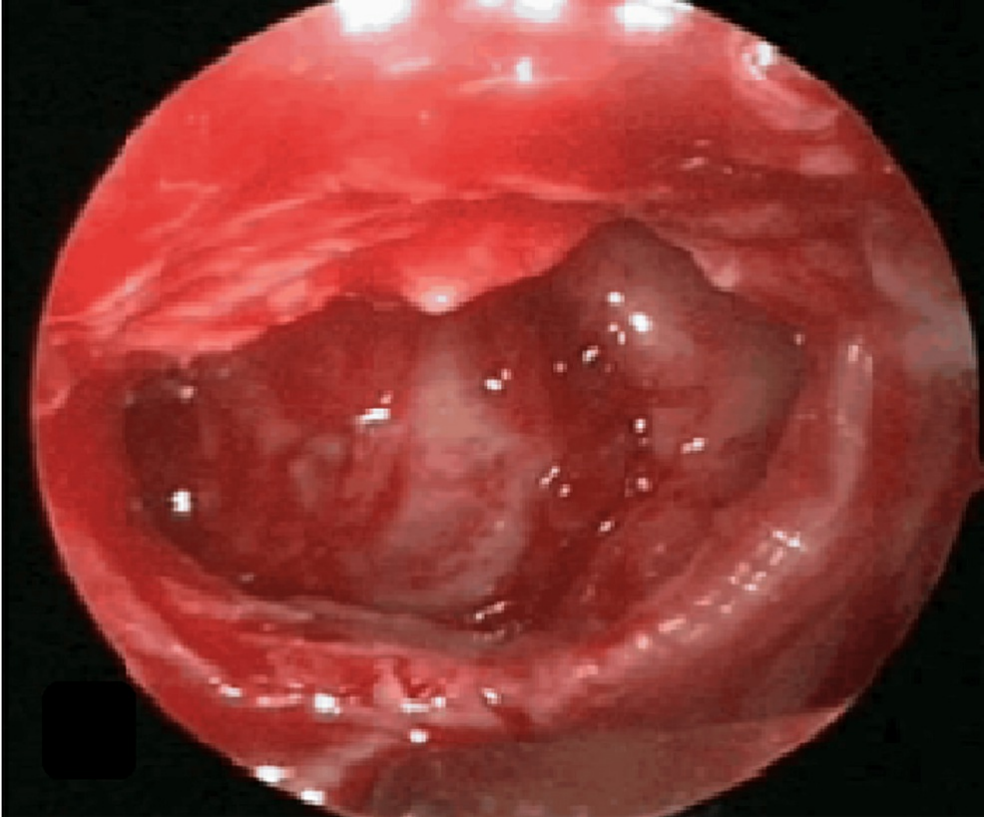
Marsupilized mucopyocele in the postoperative nasal cavity

Our surgical plan included endonasal endoscopic sinus surgery with marsupialization, which included left uncinectomy and left total ethmoidectomy. Aspiration was undertaken from the swelling, which revealed pus, confirming it to be mucopyocele. A microdebrider was used to locate and open the mucopyocele's inferio-medial wall, which had grown into the left ethmoid sinus and blocked the frontal recess. The anterior wall of the mucopyocele, which extended laterally up to the lamina papyracea, was debrided. The thick yellowish secretions of the mucopyocele were suctioned out, followed by irrigation with saline using a curved suction. After that, the mucocele cavity was examined under direct vision, and no pulsating secretions were noted (Figure 5). The left maxillary sinus ostium was widened to ensure adequate frontal sinus drainage and prevent a recurrence. 

**Figure 6:**
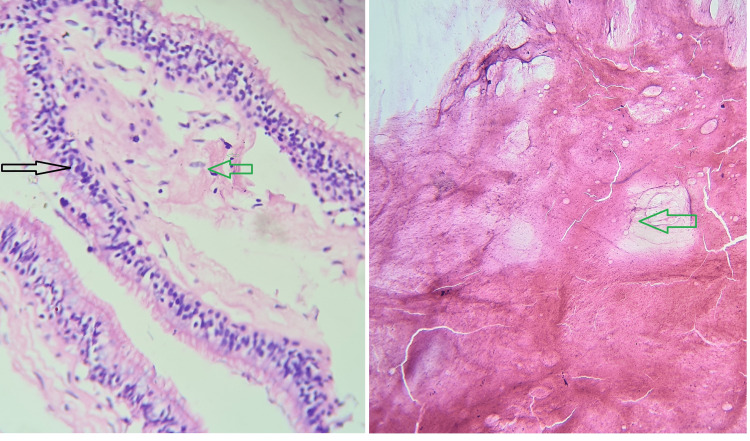
Histopathological examination: pseudostratified ciliated cylindrical epithelium (sinus mucosa, black arrow) and fibrous connective tissue (green arrow)

Histopathological evaluation (Figure 6) further showed a cystic cavity lined by a thin membrane made up of pseudostratified ciliated cylindrical epithelium suggestive of frontal mucocele.

In addition to the surgical intervention, the patient was administered 1.2 g of amoxicillin injection with clavulanate every 12 hours for seven days and 400 mg of metronidazole every eight hours for at least 72 hours, as well as analgesic and anti-inflammatory drugs. A postoperative nasal endoscopy was performed to guarantee complete disease clearance and sinus drainage. The patient was started on once-daily saline nasal irrigations with a monthly follow-up. After three months of follow-up, the patient was re-evaluated, and during the endoscopic examination, the left maxillary frontal and ethmoidal sinus ostium was found to be widely patent, with improved mucosal edema and purulent outflow showing no signs of recurrence.

## Discussion

A mucocele is an epithelium-lined sinus with a completely blocked outflow tract, where mucoid discharge is retained. The epithelial lining continues to exude mucus into this enclosed region, which leads to their propensity for growth and destruction of the surrounding tissues [1,2]. A mucopyocele is a mucocele that has contracted an infection. Paranasal sinus mucoceles are most common in the third or fourth decades of life, with a slight male predilection. The frontal sinus is the most commonly involved area (60-65%), followed by the ethmoidal sinuses (20-30%). The maxillary sinus accounts for 10% of mucoceles, whereas the sphenoid sinus is infrequently implicated (2-3%) [1-3]. Cyst development is frequently brought on by inflammation, trauma, or tumor displacement of the sinus outflow pathways [4]. Mucosal gland cystic dilatation or polyp degeneration are the essential causes, although previous sinus surgery or face trauma can be secondary causes. Cranial dysplasias, persistent sinusitis, and systemic diseases causing sinonasal symptoms are additional risk factors. Symptoms can be characterized as nasal, facial, orbital, or neurological, depending on the location of the mucocele, of which orbital symptoms are the most frequent. Because the thin-walled lamina papyracea can be moved into the optic canal by a growing mucocele, visual impairment is more prevalent with posterior ethmoid and sphenoid mucoceles [5]. The patient is more likely to experience considerable morbidity due to the paranasal sinus mucoceles' proximity to the orbit and skull base [6]. Radiographically, CT scans provide basic anatomical detail of the mucocele, delineate its interaction with neighboring bony structures, and aid in surgical planning. Bony destruction is uncommon, although bone extension and remodeling are typical in mucocele patients [7]. MRI is superior in determining the link of the mucocele to nearby soft tissue and differentiating it from other soft tissue neoplasms. Histopathologically, mucoceles resemble respiratory mucosa with regions of granulation tissue, reactive bone formation, bleeding, and fibrosis [8]. Our patient presented with an unusual symptom of progressive swelling across the medial portion of the left eye, as well as an infected mucocele and atrophic rhinitis.

To standardize frontal sinus mucocele assessment and treatment, the following classification was devised [9].

Type 1: Limited to frontal sinus (with or without orbital extension)

Type 2: Fronto-ethmoid mucocele (with or without orbital extension)

Type 3: Erosion of the posterior sinus wall:

A. Minimal or no intracranial extension 

B. Major intracranial extension

Type 4: Erosion of the anterior wall

Type 5: Erosion of both anterior and posterior walls:

A. Minimal or no intracranial extension 

B. Major intracranial extension

Surgical excision is the treatment of choice, and early intervention is indicated to prevent visual compromise in cases of mucocele [6]. Surgical excision is the conventional form of treatment, which includes an intra-nasal endoscopic approach or an external approach such as a Caldwell-Luc, osteoplastic frontal flap, or external fronto-ethmoidectomy. However, endoscopic procedures are gaining popularity with minimal rates of morbidity, recurrence, and complications in the majority of instances; it has been demonstrated to be extremely safe, successful, and effective in the clearance of the mucocele [8].​​​​ Surgical removal of the cyst in its entirety with marsupialization of the cystic wall was performed in our patient, and drainage from the frontal sinus was assured of avoiding recurrence. The patient's outcome was satisfactory, with no signs of optic neuropathy or visual impairments, as these are the most significant concerns during the recovery phase. The overall recurrence rate varies according to the source, although it is estimated to be fewer than 10% of documented cases. Poorer outcomes are linked to the posterior ethmoid and sphenoidal mucoceles and pyoceles that are not treated promptly [10].

## Conclusions

We discussed the case of a patient who presented with unusual signs and symptoms, and judicious intervention with different diagnostic modalities helped us to reach the diagnosis. As long as proper views of the paranasal sinuses are taken, the typical radiological findings of a mucocele are of tremendous use in establishing a diagnosis. The treatment for asymptomatic uncomplicated frontal mucoceles should be carried out with endoscopic sinus surgery and marsupialization. The prevention of recurrence also depends on ensuring adequate drainage of the involved sinus. More radical approaches are required if the mucoceles are extensive and if there is considerable bone erosion causing orbital or intracranial complications.
